# Cardiovascular and bone health of former elite infantry soldiers at middle life

**DOI:** 10.1186/2054-314X-1-3

**Published:** 2015-01-27

**Authors:** Yael Milgrom, Charles Milgrom, Naama Constantini, David Lavi, Victor Novak, Aharon Finestone

**Affiliations:** 4 Department of Internal Medicine, Hadassah University Hospital, Mt Scopus, Jerusalem, 91240 Israel; 5 Hebrew University Medical School, Ein Kerem, Jerusalem, 91120 Israel; 6 Department of Orthopaedic Surgery, Hadassah University Hospital, Ein Kerem, Jerusalem, 91120 Israel; 7 Hadassah University Hospital Ein Kerem, Jerusalem, 91120 Israel; 8 Clinical Research Center, Soroka University Medical Center, P.O.B. 151, Beer-Sheva, 85025 Israel; 9 Assaf HaRofeh Medical Center affiliated to The Sackler School of Medicine, Tel Aviv University, Tzrifin, 70300 Israel

**Keywords:** Cardiovascular risk, Cortical bone width, Middle age, Elite infantry soldiers

## Abstract

**Background:**

The long term implications of elite infantry service on cardiovascular health and cortical bone width at middle age has not been studied. The purpose of this study was to compare the cardiovascular health and cortical bone thickness of former elite infantry soldiers at middle life with a sedentary population of religious scholars using seven factors associated with cardiovascular risk and QCT tibial cross sections 8 cm above the ankle joint.

**Results:**

Torah scholars had a higher 5 year risk for a fatal or non fatal cardiovascular event (p = 0.008) than former elite infantry soldiers. The former elite infantry soldiers had wider tibia cortices than Torah scholar (p = 0.003).

**Conclusions:**

This study shows that former elite infantry soldiers who performed strenuous physical activity during their military service and continued physical activity in their subsequent life, had stronger tibias based on increased cortical width and a modest decrease in cardiovascular risk at middle life compared to sedentary Torah scholars.

## Background

Within the population of combat soldiers, special forces soldiers undergo the most demanding physical training and physical duties during their military service. Their activity is well beyond the exercise recommendations of the American Surgeon General [1] for maintenance of cardiovascular heath. His recommendations are to perform at least 150 minutes of mild to intensive exercise a week.

The American Heart Association recommends that the general population meet seven metrics to improve cardiovascular health: not smoking, be physically active, have normal blood pressure, glucose, total cholesterol values, and weight and eat a healthy diet [2]. Exercise capacity and activity status has been found to be related to the risk of cardiovascular and overall mortality. This effect is skewed, with the most striking difference in mortality rate being between the least fit and the next to the last fit quintile [3]. De-conditioning of cardiovascular fitness has been shown to occur rapidly. Endurance exercise trained subjects have a 16% decrease in maximum oxygen uptake 56 days after stopping training, and the decline continues unless training is resumed [4].

The risk for a fatal or non fatal cardiovascular event of former elite infantry soldiers at middle life is not known. Their training differs from that of elite athletes. Unlike athletes, they are not privileged to change their training according to their individual needs or desires. They often train and perform their duties while sleep deprived and/or nutritionally deprived. It is not known if the demanding service of the elite infantry soldier has any long term detrimental cardiovascular effects.

The Israeli population offers a unique model to study the bone and cardiovascular health at middle age of former elite infantry soldiers. The Jewish population is subject to compulsorily military service at age 18 and is composed of a closely related genome [5]. Prior to induction subjects are given a health profile based on a comprehensive physical exam and review of their health records. Subjects who receive high profiles can volunteer for elite infantry units. Since the 1980’s the databases of prospective studies involving overuse injuries among elite infantry soldiers are preserved. Army service may be deferred for those who study the Torah. Most Torah students continue the sedentary life style throughout their life. They represent a population who do little physical activity. Previously Torah scholars who had health profiles that allowed them to volunteer for elite infantry service formed the control group in longitudinal studies assessing the health of the knees, lower backs and weight bearing bones of former elite infantry soldiers at middle life [6, 7]. In this study we use the same model to evaluate the effect of the extreme physical activity of former elite infantry soldiers and their subsequent life style on their cardiovascular health and their tibia cortical bone width at middle life.

## Methods

In 1983, 295 elite male Israeli infantry recruits were part of a prospective study of stress fractures, beginning in infantry basic training [8]. For the purposes of the current study a cohort of former soldiers was assessed 25 years later. The control population was comprised of Torah scholars who were eligible for induction into the army in the period of 1981 to 1985, but did not serve. These Torah scholars had army health profiles which allowed them to volunteer for elite combat units.

### Study group 1 (Elite infantry recruits)

A list of subjects from the 1983 study group who completed three years of compulsory military service as elite infantry soldiers and who continued to serve at for the next seventeen years in the reserves as combat soldiers was obtained from the Israeli Defense Forces main computer. From this list of 51 subjects, 26 randomly chosen subjects were contact by one of the authors (C.M.) to participate in the study.

### Study group 2 (Ultra orthodox torah scholars)

A list of subjects from Jerusalem who received military health profiles which would have allowed them to volunteer for elite combat unit, whose scheduled military induction in 1982–1985 was permanently deferred because they were Torah scholars and who continued to study Torah full time at least until the age of 25 was obtained from the Israeli Defense Forces. From this list of 52 subjects, 20 were contacted by a rabbi active in the medical affairs of the Jerusalem area ultra orthodox community and asked to participate in the study.

Each subject underwent the following assessments:

A general questionnaire was administered orally, detailing the subject's military service, education, Torah studies, social economic status, smoking history, medical history, and maternal and paternal origins (Ashkenazi or Sephardic). Current alcoholic consumption was assessed by the recording number of glasses of alcoholic beverages consumed weekly. The total intake was converted into units of eight ounces size with 14% alcoholic content. Smoking history was assessed by the lifetime pack years smoked. Economic status was divided into four income brackets: 1) less than $2,000/month; 2) $2000 -$4000/month; 3) $4000-$6000/month; 4) > $6000/month. The mean economic bracket was calculated for each of the cohorts.

The daily physical activity questionnaire of Aadahl and Jorgensen [9], to quantify the daily physical activity of a subject was orally administered representing an average activity day during the week. The intensity of every specific activity was scored in units of metabolic equivalents (METS). MET is defined as the ratio of the work equivalent rate of an activity to the resting metabolic rate, which is approximately 1 kcal per kilogram of body weight per hour. The questionnaire listed nine levels of physical activity, ranging from sleep (0.9 METS) to strenuous activity (>6 METS). For each activity level the MET value is multiplied by the time spent in hours for the particular activity. The MET- time value for each activity is added to obtain the 24 hour total MET time.

The semi-quantitative food frequency questionnaire (FFQ) of Shahar et al. [10] was administered orally. It represents the average weekly food intake of subjects. Dietary intake was assessed for classifications of total energy, protein, carbohydrates, fat, calcium, phosphorus, calcium, caffeine, vitamin D and fiber. This questionnaire has been validated for the Israeli population.

The sports questionnaire used in previous Israel Defense Forces studies evaluating subjects previous five years of sport activity was administered orally [11].

The following measurements were obtained:

Measurement of height and weight and calculation of body mass index (BMI);

Two measurements of abdominal girth at the level of the umbilicus with the mean used for calculations;

Two measurements of girth of abdomen at the midpoint between the iliac crest and lower border of 12th rib with the mean used for calculations.

Pulse and blood pressure measurements were taken on the right arm and right ankle with the subject lying supine using a Dinamap GE PRO 400 V2, (GE Healthcare, Chalfont ST. Giles, U.K). The ankle/brachial blood pressure index was calculated.

The following laboratory tests were performed: Hemoglobin A1C measurement using A1C Now + multi-test system (Bayer Health Care, Sunnyvale, CA);

Total cholesterol measurement using Cardio Chek PA, (Polymer Technology System Inc. Indianapolis, IN).

QCT of right tibia was performed 8 cm above the right ankle joint using a Phillips Brilliance 64 CT, (Eindhoven, Netherlands) with a calibration phantom in the field of K2HPO4 ranging from 0 to 300 mg/cm3 in five burettes. Six axial slices with a slice thickness of 1.25 without overlap were done just proximal and distal to the 8 cm point. The cross sections were divided into posterior, anterior, medial and lateral quadrants A custom program was used to calculate the tibia cortical thickness based on the mean of measurements made of the cortical thickness at each of the posterior, anterior, medial and lateral quadrants of the cross sections.

### Primary outcome measures

1. Five years risk score for fatal and non-fatal cardiovascular events at the second assessment (25 years following the time of conscription) was calculated using the non-laboratory based risk as described by Gaziano et al. [12] based in the following parameters: gender, age, BMI, systolic blood pressure, diabetes or no diabetes and smoking status for calculations.
2. Mean tibia cortical thickness 8 cm above the ankle joint.

### Ethics statement

The study was approved by both the ethics committee of the Israel Defense Forces Medical Corps and the Hadassah University Hospital and was registered with ClinicalTrials.gov (NCT00270608). Informed written consent was obtained for all participants.

### Statistical analysis

The data on continuous variables were presented as mean ± SD. Categorical data were shown in counts and percentages. Difference in categorical variables was assessed by Chi-Square for general association, Chi-Square for trend and Cochran Mantel-Haenszel Row Mean Score tests. T-tests were applied to continuous variables. Two-sided P-value of <0.05 was considered significant. The association between tibia cortical width and other variables was assessed with Pearson correlation coefficients. Statistical analysis was made using the Statistical Analysis System version 9.2 (SAS Institute Inc., Cary, North Carolina, USA).

## Results

Twenty five of twenty six former soldiers contacted agreed to participate in the study. All 20 of the subjects contacted by the rabbi agree to participate in the study. Twenty of the soldiers 80%) and 17 of the Torah scholars (85%), had both maternal and paternal Ashkenazi origins. None of the study subjects was taking medication for hypertension, dyslipidemia or diabetes. None had a history of a previous cardiovascular event.

Table 1 summarizes the general characteristics of the two study populations. Soldiers were taller and had lower resting pulses and abdominal girths Sixteen percent of soldiers and 45% of Torah scholars had abdominal girths measured at the umbilicus greater than 102 cm (p = 0.03). The mean systolic and diastolic pressures were below the 140 over 90 range for both the Torah scholars and the soldier cohorts. Four Torah scholars and two soldiers had systolic blood pressures above 140. Four of the Torah scholars (20%) and eight of the soldiers (32%) had total cholesterol values above 200, (p = 0.3). No subject in this study had an HbA1c level above 6.5% (threshold for diagnosing diabetes).

**Table 1:** **Characteristics of study populations (means ± S.D.)**

Variable	Torah scholars N = 20	Soldiers N = 25	P value
Age (years)	43.7 ± 3.3	43.2 ± .5	0.9
Years of education	15.7 ± 2.36	15.9 ± 2.03	0.9
Economic bracket (1 to 4)	1.85 ± 0.59	3.24 ± 0.83	0.000001
Packs years smoking	4.4 ± 6.2	3..1 ± 6.6	0.26
Current alcohol consumption (units/wk)	0.3 ± 0.58	2.1 ± 3.51	0.01
Height (cm)	176 ± .4.9	179 ± 3.9	0.02
Weight (kg)	85.9 ± 15.6	84.5 ± 8.38	0.7
Weight (kg in 1983)	73.0 ± 9.6	73.7 ± 5.8	0.9
BMI	27.8 ± 5.3	26.3 ± 2.74	0.3
Pulse, min^−1^	68.9 ± 7.89	61 ± 11.301	0.01
Systolic BP, mmHg	132.8 ± 10.1	127.9 ± 9.9	0.09
Diastolic BP, mmHg	77.1 ± 6.1	76.3 ± 7.5	0.73
Ankle/Brachial Index	1.14 ± 0.11	1.22 ± 0.12	0.02
Abdominal girth at umbilicus (cm)	100.7 ± 9.59	94.7 ± 7.36	0.03
Abdominal girth at midpoint 12th Rib-Iliac Crest (cm)	99.8 ± 11.6	93.9 ± 7.22	0.06
Packs years smoking	4.4 ± 6.2	3..1 ± 6.6	0.26
Current alcohol consumption (units/wk)	0.3 ± 0.58	2.1 ± 3.51	0.01
Daily physical activity (METS)	32.9 ± 6.8	43.6 ± 9.46	0.0001
VO_2_ Max 1983 Before basic training		47.8 ± 9.2	
VO_2_ Max 1983 After basic training		53.8 ± 7.5	
HbA1C	5.61 ± 0.402	5.54 ± 0.382	0.55
Total cholesterol	177 ± 35.8	171 ± 44.1	0.63

Table 2 summarizes the current dietary intact of the two groups based on the Food Frequency Questionnaire. The soldiers had a significantly higher calcium intake (p = 0.01) and fat intake (p = 0.04) than the Torah scholars. The higher fat intake of soldiers is secondary to their eating more red meat than the Torah scholars. Table 3 summarizes the five year risk for fatal and non fatal cardiovascular events of the two groups. Seventy percent of the Torah scholars and 36% of the soldiers had a risk greater than five per cent (p = 0.02) for cardiovascular events in the following five years.

**Table 2:** **Nutritional parameters from Food Frequency Questionnaire (FFQ)**

Variable	Torah scholars N = 20	soldiers N = 25	P value
Energy Intake (Kcal)	2569 ± 865	2732 ± 846	0.53
Protein (g)	116 ± 38	122 ± 38	0.55
Fat (g)	91 ± 30	112 ± 40	0.04
Carbohydrates (g)	337 ± 145	328 ± 109	0.81
Calcium (mg)	905 ± 338	1240 ± 584	0.01
Phosphorus (mg)	1735 ± 534	1983. ±686	0.19
Vitamin D (iu)	156 ± 72	151 ± 96	0.84
Caffeine (mg)	327 ± 188	329 ± 132	0.97
Fiber (g)	26 ± 11	27 ± 9	0.74

Daily intake.

**Table 3:** **Risk of five year fatal or non Fatal cardiovascular event**

5 year cardiovascular risk	Torah scholars N = 20	Soldiers N = 25	
<5%	6	16	
5-10%	11	9	
10-20%	2	0	
20-3-%	1	0	

P for trend = 0.008

Calculated with non Laboratory Based Model.

Table 4 summarizes the difference in the mean tibia cortical thickness of tibia at the level 8 cm above the ankle joint. The solders had significantly thicker tibia cortices at the measured level than Torah scholars (p = 0.0016). The Pearson correlation coefficients between tibia cortical width and weight (0.09) and height (0.132) were very low and not significant.

**Table 4:** **Mean tibia cortical width at a cross section 8 cm above the ankle joint**

	Torah scholars N = 20	Soldiers N = 25	P value
Tibia cortical width	4.239 ± 0.65	4.869 ± 0.61	0.002

## Discussion

This project was designed to complete the assessment of the health status of former elite infantry soldiers at middle life by ascertaining their cardiovascular risk for a fatal or non-fatal event of compared to a sedentary control group. The former elite infantry soldiers were found to have a lower cardiovascular risk than the sedentary controls. No evidence was found of a detrimental effect of their demanding training as elite infantry soldiers on their cardiovascular status in middle age. Overall, their military service combined with their subsequent life styles was found to be related to better cardiovascular health.

The cardiovascular risk for a fatal or non-fatal event can be calculated on the basis of laboratory based data or non-laboratory based data. The non-laboratory based risk as described by Gaziano et al. [12] uses the parameters of gender, age, BMI, systolic blood pressure, diabetes or no diabetes and smoking or non smoking for calculations. For men, it has been shown to be as accurate a predictor of cardiovascular risk as laboratory based data. Using this method 70% of the Torah scholars were found to be at a >5% risk for a cardiovascular event in the next 5 years as compared to 36% in the soldiers cohort (p = 0.02).

Seven factors associated with risk for cardiovascular disease were assessed in this study: abdominal girth, BMI, blood pressure, resting pulse, ankle brachial pressure index, total cholesterol and diabetes status. For three of these factors: abdominal girth, resting pulse and ankle brachial index a statistically significant difference was found between the two study groups, with Torah scholars having values that might be predictive of the increased CV risk. In addition, there was a statistical trend for a higher systolic blood pressure among the Torah scholars.

Torah scholars were found to have greater abdominal girths than the soldiers. Current guidelines propose a waist circumference in males of 102 cm as the cutoff point defining abdominal obesity and increased risk for disease [13]. Forty five percent of Torah scholars, and sixteen percent of soldiers had abdominal girths measured at the umbilicus greater than 102 cm. A positive association between waist circumference and the risk for cardiovascular and all cause mortality is well established [14]. This association has been confirmed in different settings and populations [15].

The resting pulse rate has been found to be associated with increased cardiovascular mortality. Kizilbash et al. [16] showed that a resting pulse less than 75 beats per minute when compared to a resting pulse above 85 beats per minute in males aged 18–39 was associated with lower cardiovascular mortality in a 32-year follow-up. Although the mean resting pulse of Torah scholars (68.9 beats per minute) was significantly higher than those of soldiers (61 beats per minute) in this study, both of these values placed them in the decreased risk group according to the Kizilbash study. Cooney et al. [17] found a strong graded relationship between resting heart rate and cardiovascular disease in both men and women.

The ankle brachial index has been used for years as a quick and simple means to assess peripheral vascular disease in the lower extremities [18]. An index of 0.9 or less indicates an increased risk for cardiovascular disease [19]. In this study the mean ankle brachial pressure index of soldiers was found to be significantly higher than that of Torah scholars, but the lowest index found in this study was one, meaning that by this criteria none of the participants were at increased risk for cardiovascular disease.

The American Heart Association defines hypertension as 140 over 90 or higher.

Carretero and Oparil [20], state that there is no specific level of BP where cardiovascular and renal complications start to occur; thus the definition of hypertension is arbitrary. In this study none of the subjects were treated for hypertension. There was however, a statistical trend toward a higher mean systolic blood pressure among the Torah scholars, 133 mm versus 128 mm (p = 0.09). Cutler [21], in a review of selective prospective observational studies found that elevated systolic pressure is a more powerful predictor of cardiovascular events than diastolic pressure and the relationship is graded.

The current dietary intake of the cohorts was similar in this study, although solders had 27% higher fat and 37% higher calcium intakes, but there was no difference in the smoking history between the cohorts. The higher fat intake of soldiers was because they eat more red meats. There was no statistical difference found in the mean total cholesterol of the cohorts, which was measured by a quick test. Because this test did not measure a full lipid profile, the cardiovascular risk could not be calculated using the Framingham score. It also should be noted that the current dietary intakes of study subjects does not necessarily reflect their last 25 years of eating behavior.

The elite infantry soldier cohort in this study were part of a 1983 prospective study of stress fractures during their infantry basic training [8]. A 31 percent incidence of stress fractures was found, reflecting the difficulty of that training. The majority of stress fractures were of the tibia. If adaptive bone strengthening in response to physical load occurs, it can be expected to be seen in the tibia of the soldier cohort. In this study the tibia cortical bone width was measured at a point 8 cm above the ankle joint. Soldiers were found to have wider tibia cortices than Torah scholars. Height and weight are physical parameters that might be thought as parameters related to tibia cortical width. The soldier cohort was taller than the Torah scholars. However the correlation coefficients between tibia cortical width and subject height and weight were very low in this study. Therefore the observed difference in the tibia cortical width of the cohorts can be explained either by an adaptive strengthening response of their bone to loading or to an unidentified selection bias in the groups.

The soldiers in the present study served for a minimum of three years as elite infantry soldiers, and afterwards continued to serve each year in the army reserves as elite infantry soldiers for the next 17 years. At the time of the current study, 25 years after the induction of the soldier cohort into the army, it was found that the soldier cohort continued to do much more physical activity than the Torah scholars. They burned 1/3 more calories daily than did the Torah scholars, who largely see exercise as a waste of studying time.

A strength of this study is that the cohorts came from similar ethnic backgrounds. Most were Askenazi with a minority of Sephardic Jews. Both of these groups have been shown to have a genome which is closely related. In a Hungarian twin cohort study factors associated with cardiovascular risk such as weight, waist circumference and blood pressure where found to be highly associated with genetics [22]. Second, all of the subjects in this study had comprehensive medical reviews at the age of 17. All of them received health profiles at that time which allowed them to volunteer to service as elite infantry soldiers. Third, the smoking history of the cohorts was similar and no subject drank more than one glass of wine or beer a day. A weakness of the present study is that the number of subjects in each cohort is relatively small and that no individual measurement of high and low density cholesterol was made. Additionally there may have been a pre-selection of subjects with better physical capabilities in the elite infantry cohort.

This study found that the cardiovascular risk status at middle life of former elite infantry soldiers was modestly better than that of sedentary Torah scholars. While the soldier cohort reached the level of good cardiovascular fitness during basic training with a mean VO_2_ Max of 53.8, deconditioning would have occurred rapidly when they finished their army service unless they continued regular cardiovascular training. Therefore, the soldiers’ current cardiovascular risk is likely to be related to their life style after they completed army service. The soldiers difficult training did have a long lasting effect on their tibia strength. Their tibia cortical width was 15 percent higher than that of the Torah scholars. We previously reported that the former elite infantry soldiers in this study cohort had stronger tibias than the Torah scholars based on another geometric property, an increased area moment of inertia of the tibia [6]. No difference in the bone mineral content of the tibia cortex between the cohorts was found in these studies. There was also no difference in the health status of the knees between the two cohorts, judged by MRI criteria, but the former elite infantry recruits had more degenerate lumbar disk changes by MRI criteria [7].

## Conclusion

The findings of this study when combined with previous published studies from the same cohorts shows that the extremely demanding physical training of the elite infantry recruit is not detrimental to their health at middle life and in some aspects may even be beneficial. This knowledge is important both to those who are recruited to serve in elite infantry units and to those who send them to serve in such units.

## Authors’ information

I.D.F. Medical Corps active reserve: Yael Milgrom, Charles Milgrom and Aharon Finestone.
